# Genomic Evidence for the Rise of *Salmonella* Typhimurium ST34 with Increased Plasmid-Mediated Resistance in the Thailand Pork Chain

**DOI:** 10.3390/pathogens14121190

**Published:** 2025-11-21

**Authors:** Hongmei Liu, Ning Wang, Sunpetch Angkititrakul, Wengui Li, Zhongyang Luo, Mingpeng Hou, Yi Wu, Yubo Shi, Yuelin Wang, Fengyun Li, Yaowen Liu, Xin Wu, Fanan Suksawat

**Affiliations:** 1 Faculty of Veterinary Medicine, Khon Kaen University, Khon Kaen 40002, Thailand; liuhongmei.ll@hotmail.com (H.L.); sunpetch@kku.ac.th (S.A.); 2 Yunnan Joint International R&D Center of Veterinary Public Health, College of Veterinary Medicine, Yunnan Agricultural University, Kunming 650051, China; 3 Mengla County Animal Husbandry and Veterinary Station, Xishuangbanna Prefecture, Jinghong 666106, China; 4 School of Life Sciences, Xishuangbanna Vocational and Technical College, Menglun 666300, China

**Keywords:** *S.* Typhimurium, ARGs, plasmid, mobility types, pork chain, One Health, Thailand

## Abstract

Background: Mobile antimicrobial resistance genes (ARGs) on plasmids or other elements enable *Salmonella* Typhimurium to spread resistance across hosts and environments. The emergence of multi-drug resistance (MDR) *Salmonella* Typhimurium has raised global concern, yet little is reported about these mobile elements from the Thailand pork supply chain, where this risk of transfer to humans remains largely uncharacterized. Methods: Between March 2023 and February 2024, 25 *S.* Typhimurium isolates were collected from pig carcasses in slaughterhouses and pork swabs from retail markets in northeastern Thailand. Nine representative isolates, sampled across three seasons, were subjected to Illumina whole-genome sequencing. Assemblies were analyzed for sequence types, phylogenetic relationships, antimicrobial resistance (AMR) determinants, plasmid replicons and mobilization features, functional annotation based on COG (Clusters of Orthologous Groups of proteins) classification, and comparative genomics against a reference strain. Results: Genome assemblies ranged from 4.76 to 5.00 Mb with consistent GC (guanine-cytosine) content (52.0–52.2%). Phylogenetic analysis revealed three sequence types: ST34 (77.8%), ST19, and ST1543. ST34 isolates displayed the broadest AMR gene repertoires, carrying tetracycline (*tetA/tetB*), sulfonamide (*sul1/sul2/sul3*), aminoglycoside (*aadA*, *aph(6)-Id*, *aph(3″)-Ib*), phenicol (*floR*, *catA1*), and β-lactam (*bla_TEM-1B*) genes, whereas non-ST34 isolates harbored fewer determinants. ARGs frequently co-localized with *IncQ1* and *Col-*type plasmid replicons, *MOB_H/MobA* relaxases (enzymes that initiate plasmid transfer), and conjugation modules (type IV secretion and coupling proteins), often alongside virulence loci and metal resistance operons. Functional annotation showed highly conserved metabolic and housekeeping functions, while comparative genomics confirmed >90% core genome conservation, with variability concentrated in genomic islands encoding hypothetical proteins. These genomic patterns were inferred from a limited WGS dataset (nine isolates) and should therefore be considered exploratory and require confirmation in larger collections. Conclusions: Multi-drug resistant ST34 *Salmonella* Typhimurium predominated in the northeastern Thailand pork supply chain, with diverse resistance genes carried on *IncQ1/Col-*type plasmids linked to *MOB_H* relaxases and conjugation modules. The stability of these mobilizable elements underscores their role in sustaining MDR traits and highlights the risk of foodborne AMR transmission, reinforcing the need for continuous genomic surveillance under a One Health framework.

## 1. Introduction

*Salmonella* enterica serotype Typhimurium (including the monophasic variant *S.* 4,(5),12:i:-) is one of the most common foodborne pathogens worldwide, causing gastroenteritis and invasive infections [1]. This has been repeatedly linked to pork production chains, reporting swine as a critical reservoir for human infection [2,3]. In recent years, the concern has shifted from simple prevalence to the growing role of antimicrobial resistance (AMR), particularly mobile determinants carried on plasmids and other genetic elements that enable horizontal transfer across bacteria and hosts. Such mobility accelerates the spread of resistance in food production systems [4], increasing the risk that consumers may be exposed to multidrug-resistant strains through contaminated pork and underscoring a major public health threat.

Globally, the epidemiology of *S.* Typhimurium has changed markedly over the past two decades. Multilocus sequence typing (MLST), which classifies bacterial isolates based on allelic variation in seven housekeeping genes, has been widely applied to track the evolutionary dynamics of *Salmonella* and to define its major clonal lineages [5]. Within this framework, sequence type (ST) 34 has expanded rapidly, often replacing the traditionally dominant ST19 [6]. Several groups, including Zhang et al. in China and Ali et al. in Korea, reported that ST34 is now the major clone in livestock, displaying high levels of MDR [7,8]. Similarly, multiple studies have shown that monophasic ST34 has disseminated widely in both Europe and Asia, frequently carrying resistance determinants to *tetracyclines* [9,10], *sulfonamides*, and *β-lactams*. In contrast, ST19 persists in more limited niches and usually harbors fewer AMR determinants. These observations supported the hypothesis that the success of ST34 is partly driven by the acquisition of mobile resistance genes, enabling its persistence in intensive animal production systems.

Beyond clonal expansion, the role of plasmids and mobilization modules has drawn increasing attention. Previous studies in Europe and East Asia demonstrated that *IncQ1-*, *Col-*type, and *IncX1* replicons frequently harbor resistance determinants [11] and are often associated with relaxases such as *MOB_H* or *MobA*, together with conjugation machinery. Many researchers emphasized that plasmids not only transmit AMR but also often co-carry virulence loci and heavy-metal resistance operons [12]. This probably provides co-selection advantages in farm environments [13]. Moreover, comparative genomics from Italian and Chinese swine production revealed recurrent co-localization of ARGs with type IV secretion systems (T4SS), underlining the high transfer ability potential of these plasmids [14]. However, plasmid structures and AMR cargo can vary between regions and production systems, highlighting the need for country-specific genomic investigations, particularly in settings where pork is a major protein source.

In Thailand and neighboring countries such as Laos and Cambodia, surveillance of *Salmonella* has primarily focused on prevalence studies and phenotypic antimicrobial susceptibility testing. For example, Prathan et al. investigated genetic diversity through serotyping of *Salmonella* combined with phylogenetic tree analysis [15]. Ananchaipattana et al. reported widespread tetracycline and ampicillin resistance among retail meat isolates [16]. Yet these studies lacked genome-level resolution, leaving plasmid architectures, mobilization modules, and the co-localization of AMR genes with other adaptive cargo largely unexplored. This revealed one critical knowledge gap, since genomic data are essential for tracing horizontal gene transfer, predicting transferability, and assessing the true public health risk of AMR spread from food sources to humans.

To address this gap, we applied whole-genome sequencing and comparative genomics to *S.* Typhimurium isolates collected from the pork supply chain in northeastern Thailand during 2023–2024. Our analysis focused on the mobilome and AMR gene co-localization patterns, with particular emphasis on ST34 *S.* Typhimurium. Integrating phylogenetic analysis, resistome profiling, plasmid annotation, functional categorization, and comparative genomics, this study aimed to characterize the genomic features of *S*. Typhimurium circulating in the pork production chain, highlighting phylogenetic relationships, resistome diversity, and the co-occurrence of AMR genes with plasmid and mobilome elements. We hypothesized that MDR ST34 isolates in the Thai pork chain would carry mobilizable plasmids co-localizing ARGs with virulence and metal-resistance loci, distinguishing them from other local *S.* Typhimurium lineages. By providing the first genome-level description of these features in Thailand, our work is expected to generate country-specific evidence to support One Health AMR surveillance and risk-based interventions along the pork supply chain.

## 2. Materials and Methods

### 2.1. Bacterial Collection and Study Design

Between April 2023 and February 2024, a targeted surveillance of the pork production chain in northeastern Thailand was conducted, yielding 897 samples collected from slaughterhouses and retail markets across three climatic seasons (summer, rainy, and winter); the slaughterhouses operated standardized processing lines with good hygiene management, following biosafety guidelines and showing no visible insects or waste. Around 20 retail vendors handled pork sales in open or semi-enclosed markets, which were generally clean and well maintained, with no large gatherings of flies or mosquitoes, though no formal disinfection schedule was recorded. Sampling was performed twice per season at slaughterhouses and twice per season at retail markets. Samples from slaughterhouses and retail markets included carcass, fecal, and pork swabs. Primary processing, presumptive identification, and isolate archiving were performed at the regional reference laboratory. *Salmonella* isolation followed the ISO 6579:2002/AMD 1:2017 (International Organization for Standardization. (2017). Microbiology of the food chain—Horizontal method for the detection, enumeration and serotyping of Salmonella—Part 1: Detection of Salmonella spp. (ISO 6579-1:2017). Geneva, Switzerland: ISO.)procedure, involving pre-enrichment in buffered peptone water, selective enrichment in modified semisolid Rappaport-Vassiliadis medium, and plating on xylose lysine deoxycholate and Hektoen enteric agars. Confirmed isolates were obtained within approximately one week, and 25 were identified as Salmonella enterica serotype Typhimurium (including 17 monophasic variants) and stored at −80 °C in glycerol stocks. Due to financial and logistical constraints, whole-genome sequencing was performed on a subset of nine isolates. These isolates were selected to maximize diversity in temporal distribution, sample origin (carcass, feces, pork), and phenotypic characteristics, and to ensure sufficient DNA quality for sequencing. Given the limited number of sequenced isolates, the WGS dataset is not statistically representative of all S. Typhimurium circulating in the study area; accordingly, downstream genomic analyses are descriptive and exploratory in nature and should not be overgeneralized to the wider population. Nine representative isolates were selected for whole-genome sequencing based on temporal distribution, sample origin, and DNA quality, while the remaining isolates were retained for potential follow-up.

### 2.2. DNA Extraction and Whole-Genome Sequencing

Genomic DNA was extracted from overnight cultures of each selected isolate using the Takara MiniBEST Bacteria Genomic DNA Extraction Kit (Takara Bio Inc., Shiga, Japan), following the manufacturer’s instructions. DNA concentration and purity were determined using a NanoDrop spectrophotometer (Thermo Fisher Scientific, Waltham, MA, USA) and Qubit fluorometer (Invitrogen, Carlsbad, CA, USA). Sequencing libraries were prepared with the Illumina DNA Prep kit and subjected to paired-end sequencing (2 × 150 bp) on an Illumina NovaSeq 6000 platform at [Shenggong Bioengineering (Shanghai) Co., Ltd, Shanghai, China]. Raw sequencing reads were quality-checked using fastp v0.11.2 to remove adapters and low-quality bases. De novo assemblies were generated with SPAdes v3.5.0, and assembly quality was assessed with QUAST v5.2. Assembly statistics, including contig number, N50, genome size, and GC content, were recorded.

### 2.3. Functional Annotation and Comparative Genomics

Predicted coding sequences (CDSs) from each assembled genome were functionally annotated using Prokka v1.14.6. COG classification was performed with eggNOG-mapper v2 against the eggNOG v5.0 database, assigning CDSs to functional categories to evaluate metabolic capacity, housekeeping functions, and defense-related mechanisms across isolates. CDS counts were summarized per category to assess inter-strain variation and overall functional distribution.

For comparative genomics, the nine *Salmonella* Typhimurium genomes were aligned against the reference strain SA606 (4,782,988 bp; GenBank accession: CP133419.1). Whole-genome similarity was visualized using BLAST Ring Image Generator (BRIG v0.95), applying thresholds of 50–100% identity. Conserved and divergent regions were identified by visual inspection of concentric ring plots. Divergent loci with reduced identity (<70%) were further annotated to determine whether they corresponded to hypothetical proteins, prophage-related sequences, or genomic islands. To quantify conservation, the proportion of each genome sharing ≥70% identity with the reference was calculated. Conserved housekeeping genes (e.g., DNA repair, recombination, cell wall biosynthesis, motility) were specifically inspected to confirm core genome stability, whereas variable regions were analyzed for evidence of horizontal gene transfer or adaptive functions.

### 2.4. Genomic Characterization

Sequence types were assigned in silico using mlst v2.19, and core-genome MLST (cgMLST) was performed with chewBBACA v3.2.0. SNP-based phylogenetic reconstruction was conducted using Snippy v4.6.0 with the *S*. Typhimurium LT2 reference genome (NC_003197.2), and maximum-likelihood trees were inferred with IQ-TREE v2.1.4 using 1000 bootstrap replicates. Publicly available Southeast Asian *S.* Typhimurium genomes were included for comparative analyses.

### 2.5. Detection of ARGs, Plasmids

AMR genes and chromosomal mutations (QRDR and pmrA/pmrB) were detected using ABRicate v1.0.1 against the ResFinder and CARD databases (identity ≥ 90%, coverage ≥ 80%). Plasmid replicons were identified with PlasmidFinder v2.1, and plasmid mobility and reconstruction were performed using MOB-suite v3.0.3, allowing determination of conjugative plasmids carrying AMR determinants.

## 3. Results

### 3.1. Comparative Whole-Genome Analysis

**Figure 1:**
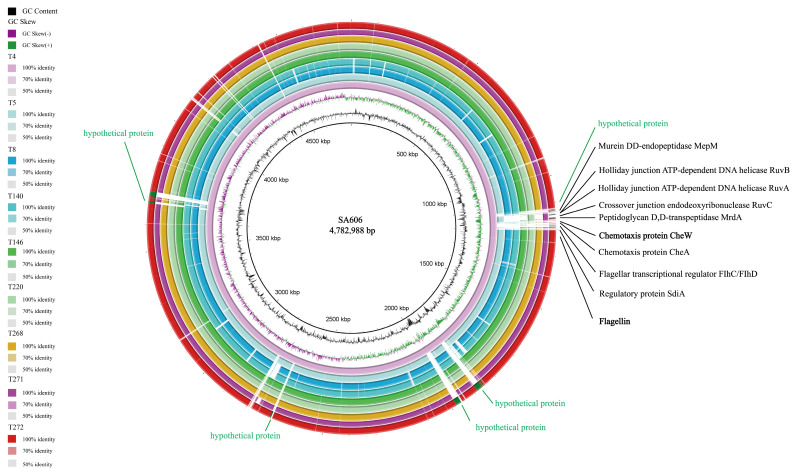
Comparative genomic analysis of the 9 *Salmonella* strains was visualized using a circular genome map (circos plot). The innermost circle shows the chromosome of SA606 with genome size and position in kbp. The next two tracks represent GC content (black) and GC skew (green and purple for positive and negative skew, respectively). The outer nine colored rings represent BLASTn similarity of each draft genome (from inside to outside: T4, T5, T8, T140, T146, T220, T268, T271 and T272) against the SA606 chromosome. For each isolate, dark shading indicates 100% nucleotide identity, intermediate shading indicates 70–99% identity and light shading indicates 50–69% identity; white gaps represent regions that are absent or highly divergent compared with the reference.

Comparative genomic analysis of the nine *Salmonella* strains against the reference strain SA606 (4,782,988 bp) revealed a high degree of overall genomic conservation, as illustrated by the concentric BLAST ring plot (Figure 1). Across all genomes, the majority of coding regions exhibited 70–100% sequence identity, with fully conserved blocks (100% identity) occupying most of the genomic backbone. Regions with reduced identity (50–70%) were limited and largely corresponded to loci annotated as hypothetical proteins, highlighting them as potential sources of genomic variability. Quantitatively, over 90% of the genome length across strains shared at least 70% sequence identity, while highly divergent regions constituted during 5–8% of the genome, suggesting that strain-specific variation is confined to discrete genomic islands. Several conserved functional genes were consistently retained across strains, underscoring the evolutionary stability of essential processes. These included genes involved in cell wall biosynthesis (murein DD-endopeptidase MepM; peptidoglycan D,D-transpeptidase MrdA), DNA repair and homologous recombination (Holliday junction helicases RuvA and RuvB; endonuclease RuvC), and motility and chemotaxis regulation (chemotaxis proteins CheA and CheW; flagellar regulators FlhC/FlhD; flagellin). Moreover, the regulatory protein SdiA, known for its role in quorum sensing and interspecies communication, was also conserved. Notably, most of the observed sequence divergence was clustered in genomic islands enriched for hypothetical proteins. These variable regions likely represent hotspots of horizontal gene transfer, phage-related insertions, or adaptive evolution, potentially conferring strain-specific ecological advantages.

In summary, the comparative analysis demonstrated that the nine *Salmonella* genomes maintain a highly conserved core genome (>90% of CDS content), particularly in housekeeping, recombination, and motility-related functions, while strain-specific differences are concentrated in ≤10% of the genome, predominantly in uncharacterized loci. This distribution suggests that functional conservation underlies common pathogenic and survival strategies, whereas variability in hypothetical protein regions may contribute to niche adaptation and phenotypic divergence.

### 3.2. Functional Annotation of Coding Sequences

**Figure 2:**
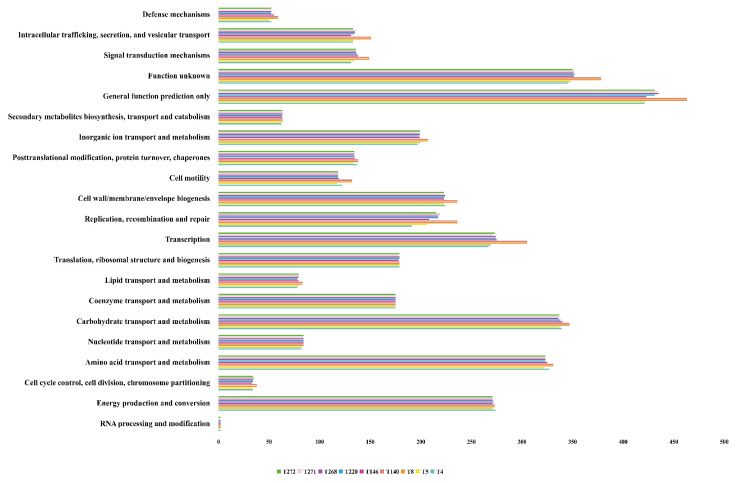
Comparison of functional categories in the 9 *Salmonella* strain genomes based on COG. Categories with particular relevance to antimicrobial resistance and virulence (defense mechanisms, cell wall/membrane/envelope biogenesis, intracellular trafficking, secretion and vesicular transport, and replication–recombination–repair) are explicitly mentioned in the text, whereas most CDSs belong to general metabolic and housekeeping categories.

COG-based functional annotation of the nine *Salmonella* genomes (Figure 2) revealed a highly conserved, housekeeping- and metabolism-biased repertoire across strains. The single largest category in every genome was “General function prediction only”, with annotated CDS counts visually estimated at 430–490 CDS per genome. The next most abundant categories were “Function unknown” (320–410 CDS) and broad metabolic classes-“Carbohydrate transport and metabolism” and “Amino acid transport and metabolism”-each comprising on the order of 300–370 CDS. Together these four categories account for the bulk of annotated coding capacity and reflect a predominance of generalist and core metabolic functions. Core information-processing categories were also prominent: “Transcription” (280–320 CDS), “Energy production and conversion” (240–280 CDS), and “Translation, ribosomal structure and biogenesis” (190–230 CDS) were consistently well represented. Categories related to genome maintenance and cell envelope were present at intermediate levels-“Replication, recombination and repair” and “Cell wall/membrane/envelope biogenesis” each at roughly 230–300 CDS-indicating substantial investment in replication/repair and surface-associated functions. “Inorganic ion transport and metabolism” similarly showed moderate representation (230–270 CDS), consistent with requirements for metal homeostasis and nutrient uptake. Functions involved in signaling and trafficking (for example, “Signal transduction mechanisms” and “Intracellular trafficking, secretion, and vesicular transport”) and protein quality control (“Posttranslational modification, protein turnover, chaperones”) were present at intermediate to lower abundances (approximately 150–200 CDS and 150–180 CDS, respectively). Specialized metabolic categories (e.g., “Secondary metabolites biosynthesis, transport and catabolism”, “Lipid transport and metabolism”, “Nucleotide transport and metabolism”) were comparatively smaller (generally <150 CDS). Notably, “Defense mechanisms” constituted only a minor fraction of the annotated CDS (visually 20–60 CDS per genome), and “RNA processing and modification” was essentially negligible in these annotations. Overall, categories with closer links to AMR and virulence (defense mechanisms, cell wall/membrane/envelope biogenesis, intracellular trafficking/secretion and replication-recombination–repair) were consistently present but represented a much smaller proportion of the coding capacity than general housekeeping and metabolic functions.

Inter-strain variation in category counts was limited: colored bars for each genome track closely across nearly all COG classes, indicating that the nine *Salmonella* genomes share a similar functional composition at the level of COG assignment. These COG profiles therefore suggest a conserved functional backbone, and imply that differences in multidrug resistance are more likely driven by specific accessory elements (e.g., plasmids and genomic islands) than by shifts in core functional categories.

### 3.3. Genome Sequencing and Assembly Quality

Illumina sequencing of the nine representative *Salmonella* Typhimurium isolates generated between 4.2 and 7.3 million paired-end reads per genome, providing high coverage suitable for downstream analyses (Table 1). After de novo assembly, the genome sizes ranged from 4.76 Mb to 5.00 Mb, consistent with the expected size of *S*. Typhimurium genomes. The overall GC content was stable at 52.0–52.2%, showing no marked deviation among isolates. The quality of assemblies was reflected in the number of contigs and N50 values. The isolate T4 yielded the most contiguous assembly, consisting of 26 contigs with an N50 of 594 kb, while T146 presented the highest fragmentation, with 74 contigs and an N50 of 223 kb. Most isolates displayed intermediate assembly quality, with contig numbers ranging from 32 to 59 and N50 values from 310 kb to 482 kb. Annotation revealed that the genomes contained 4650–4950 coding sequences (CDSs), alongside a consistent complement of tRNAs (73–76) and rRNA operons (21–23). The relatively narrow range of CDS numbers and stable distribution of functional RNAs indicate a conserved genomic backbone across the isolates.

In summary, all nine isolates were successfully sequenced and assembled with sufficient quality for comparative genomic analysis (Table 2). The assemblies displayed minor variation in genome size and contiguity but maintained highly similar GC content and functional gene composition, confirming the robustness of the dataset for subsequent phylogenetic and resistome investigations.

### 3.4. Phylogenetic Relationships and Sequence Types

**Figure 3:**
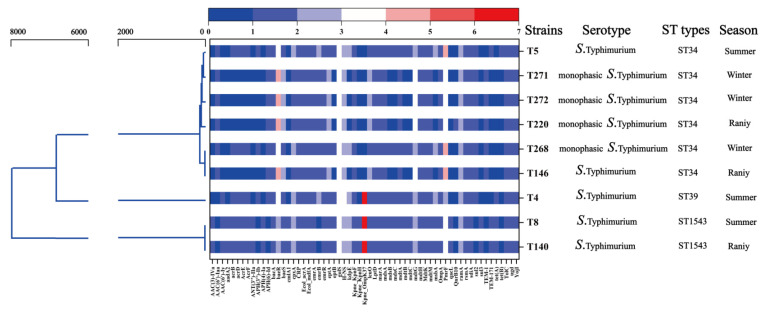
Maximum likelihood genetics of *Salmonella* isolates, heat map shown the total number of resistance genes across the entire genome. The color scale above the heatmap indicates the detection strength of each ARG, with dark blue (value 0) representing absence and warmer colors (values 1–7, shading from light blue to red) representing increasingly strong matches to reference resistance genes. Only hits with ≥80% nucleotide identity to reference sequences were retained.

Core-genome MLST and SNP-based phylogenetic analysis demonstrated that the nine *Salmonella* Typhimurium isolates formed three distinct sequence types (STs) (Figure 3). The majority of isolates (7/9, 77.8%) clustered within ST34, which represented the dominant lineage circulating across all three sampling seasons. Within this clade, both biphasic and monophasic variants were observed, suggesting ongoing diversification of ST34 in the pork supply chain. By contrast, only a single isolate each belonged to ST19 and ST1543, both of which were genetically distinct and located on separate branches of the tree. Integration of the phylogenetic framework with resistome profiling revealed that ST34 isolates carried the broadest spectrum and highest number of ARGs. The most frequently detected determinants included tetracycline resistance genes (*tetA*, *tetB*), sulfonamide genes (*sul1*, *sul2*), and phenicol resistance genes (*floR*, *catA1*), each present in the majority of ST34 isolates. Additionally, aminoglycoside resistance genes (*aadA1*, *aph(6)-Id*, *aph(3″)-Ib*) were enriched within the ST34 cluster, indicating a tendency toward multidrug resistance. In contrast, the two non-ST34 isolates (ST19 and ST1543) harbored substantially fewer AMR determinants, typically limited to three or four genes, and lacked the fluoroquinolone-associated mutations detected in ST34. Across all isolates, intrinsic efflux systems (*acrAB-tolC*, *mdsABC*) were universally present, underscoring a conserved background of multi-drug efflux capacity.

Overall, the combined phylogenetic and resistome analysis highlights ST34 as the predominant and most multi-drug resistant lineage, whereas other sequence types appear sporadically and with narrower resistance profiles. However, only two non-ST34 isolates were available for genomic analysis, which limits broader inferences about the epidemiological role of ST19 and ST1543 in this setting.

### 3.5. ARGs Profiles

**Figure 4:**
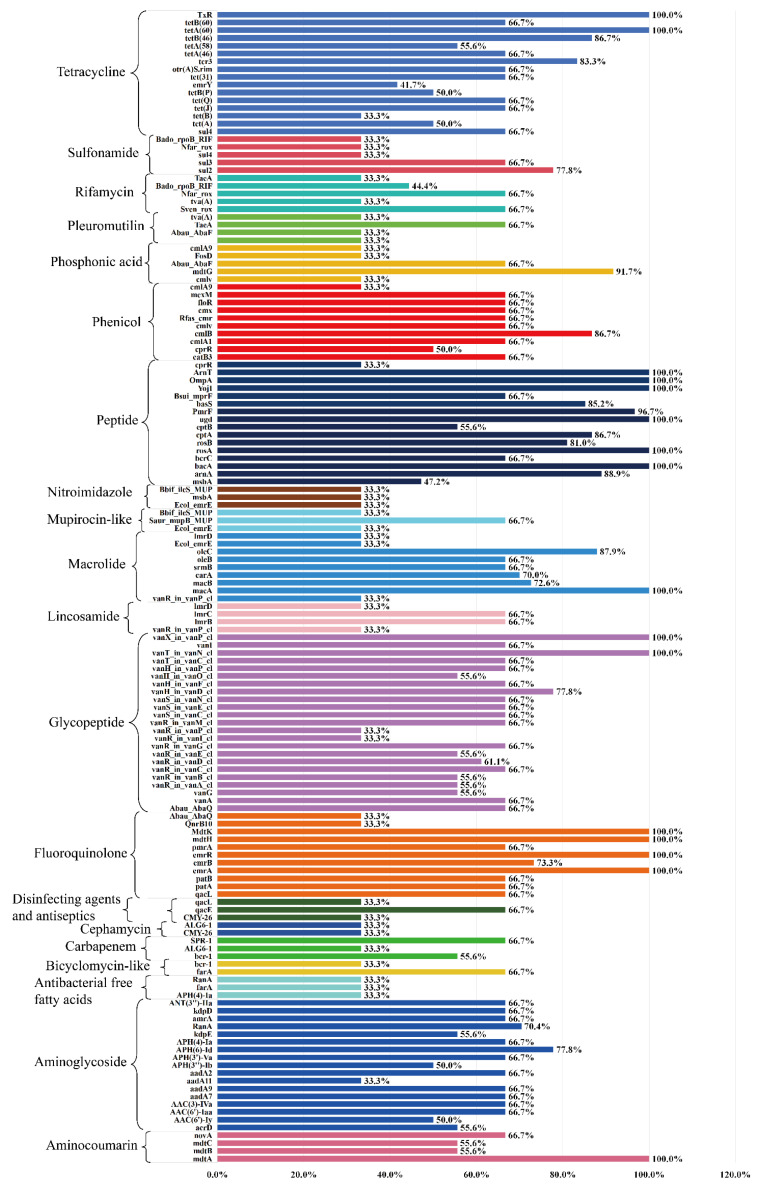
Distribution of acquired antimicrobial resistance (AMR) determinants in nine Salmonella isolates. Each horizontal bar represents the proportion of isolates carrying a given resistance determinant, with the percentage shown at the end of the bar. Genetic elements (AMR genes or associated regulators) are listed along the left axis and are grouped by their corresponding antimicrobial class (e.g., tetracyclines, sulfonamides, rifamycins, pleuromutilins, phosphonic acid derivatives, phenicols, peptide antibiotics, nitroimidazoles, macrolides, lincosamides, glycopeptides, fluoroquinolones, disinfectants and antiseptics, β-lactams, aminoglycosides and aminocoumarins). Bars are color-coded according to antimicrobial class, allowing visualization of the prevalence of each resistance determinant across the nine sequenced isolates.

Analysis of the resistome revealed a wide distribution of AMR determinants across diverse antibiotic classes. In the single-class resistance profile (Figure 4), genes conferring resistance to tetracyclines were the most prevalent, with multiple determinants (e.g., *tetR*, *tetQ*, *tetX*) exhibiting detection rates ranging from 66.7% to 100%. This was followed by resistance genes associated with aminoglycosides and glycopeptides, which also displayed high abundance and detection frequency (>70% in several loci). Moderate representation was observed for fluoroquinolone, phenicol, and macrolide resistance genes, typically ranging between 55 and 87% detection, whereas determinants of sulfonamides, rifamycins, pleuromutilins, and phosphonic acids were less common, generally below 50% prevalence.

**Figure 5:**
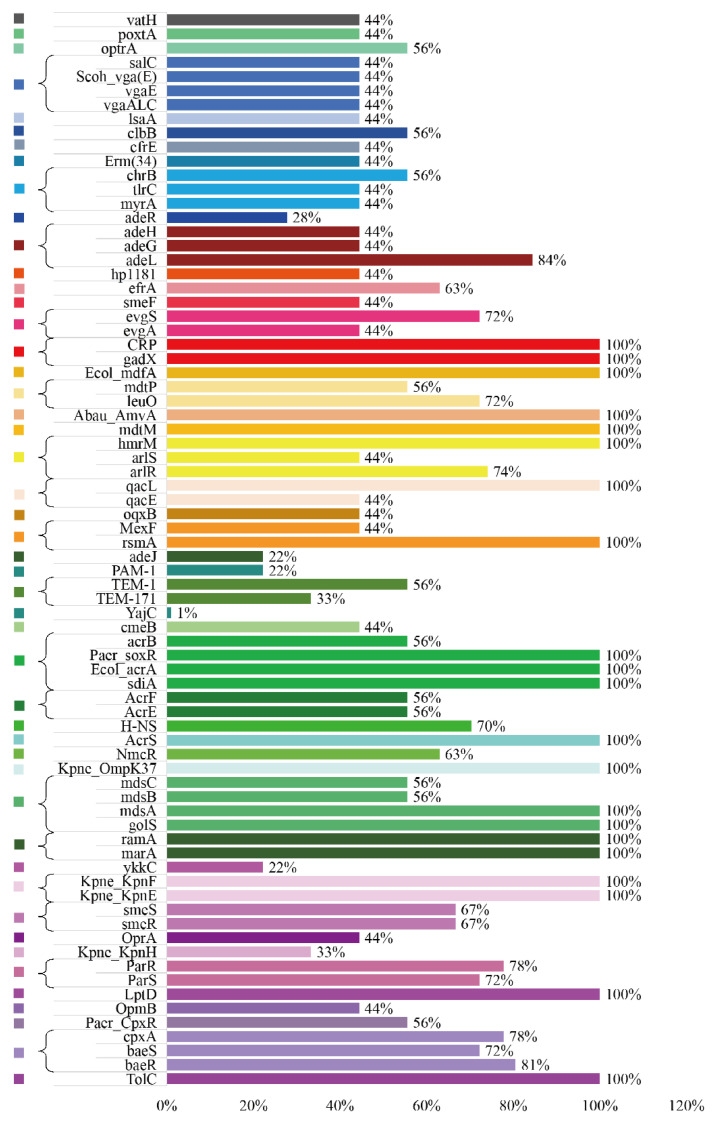

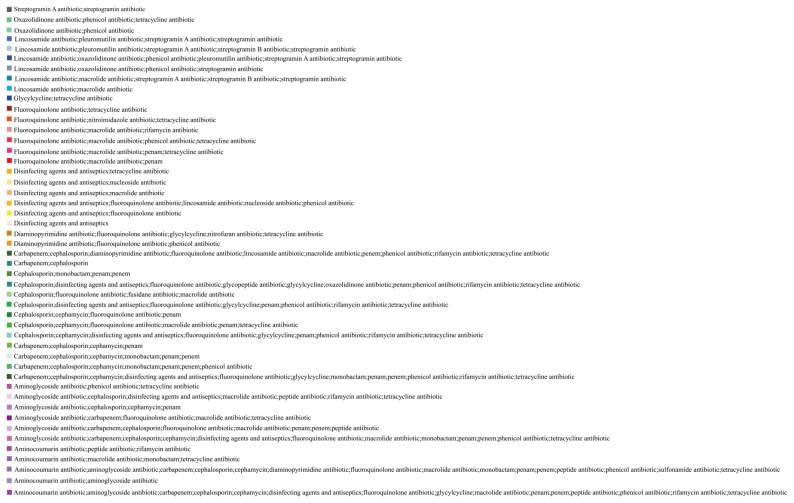
Multidrug resistance determinants and their predicted antimicrobial spectra in nine Salmonella isolates. Horizontal bars indicate the proportion of isolates in which each resistance gene or regulator was detected, with gene names listed along the left axis and percentages shown at the end of the bars. Only determinants predicted to affect more than one antimicrobial class are included. Bars are color-coded to reflect the combination of antimicrobial classes potentially impacted by each determinant (for example, macrolides–lincosamides–streptogramins, phenicols–tetracyclines, fluoroquinolones–aminoglycosides or broader multidrug spectra). The underlying drug-class annotations were retrieved from the Comprehensive Antibiotic Resistance Database (CARD) “Drug Class” ontology.

The multi-class resistance analysis (Figure 5) highlighted the coexistence of diverse AMR determinants within the same bacterial genomes. Efflux pump–associated genes, such as *acrA*, *acrB*, *mdfA*, *tolC*, and regulatory elements (*soxR*, *marA*), were widely distributed, with several reaching 100% occurrence, suggesting a central role of multidrug efflux systems in mediating broad-spectrum resistance. Notably, resistance determinants related to Enterobacteriaceae efflux systems (*acrAB-tolC* and related regulators) and fluoroquinolone/aminoglycoside modifying enzymes showed particularly high frequencies, reinforcing their contribution to multi-drug resistance phenotypes. Further functional annotation of multi-label resistance clusters demonstrated that resistance to fluoroquinolones, aminoglycosides, and β-lactams (including cephalosporins, carbapenems, and monobactams) frequently co-occurred with tetracycline and phenicol resistance genes, indicating strong co-selection patterns. In particular, aminoglycoside- and aminocoumarin-associated clusters exhibited the highest degree of multidrug overlap, often incorporating resistance determinants from more than five antibiotic classes simultaneously. Conversely, resistance clusters involving mupirocin-like or phosphonic acid remained relatively rare and did not exhibit extensive cross-class linkage.

Overall, these findings demonstrated that while tetracycline resistance is the most dominant single-class feature, multi-drug resistance is largely driven by efflux-associated determinants and the co-occurrence of *aminoglycoside*, *fluoroquinolone*, and *β-lactam* resistance genes, underscoring the complexity of resistome architecture.

### 3.6. Plasmid Replicons and Mobility Types

Annotation of nine strains (T4, T5, T8, T140, T146, T220, T268, T271, T272) revealed a highly recurrent and co-localized set of antimicrobial-resistance genes (ARGs) (Table 3) and associated mobile-element signatures (Table 4). All nine strains were annotated to carry *aac(6′)-Iaa*. The majority (7/9) additionally harbored the aminoglycoside phosphotransferases *aph(6)-Id* and *aph(3″)-Ib*; two strains (T8, T140) instead contained alternative aminoglycoside resistance determinants (e.g., *aadA2*, *ant(3″)-Ia*, *aac(3)-IVa*). Tetracycline resistance (*tet(A)* or *tet(B)*) was detected in 7/9 strains, sulfonamide resistance (predominantly *sul2*; *sul3* in T8 and T140) in 7/9 strains, and *bla_TEM-1B* in 8/9 strains. A plasmid-borne quinolone determinant (*qnrB19*) was observed only in T220. Thus, the dominant ARG constellation in this collection comprises *aac(6′)-Iaa* together with aph family genes, *tet(A/B)*, *sul2/sul3* and *bla_TEM-1B*.

Linking ARG annotations to plasmid/mobilization features showed clear co-localization trends. Six of nine strains (T4, T5, T146, T268, T271, T272) were annotated with *IncQ1* replicons; several others carried Col-type replicons (T220: Col(pHAD28); T271 additionally carried Col(BS512) and *IncX1*). A *MOB_H-*type relaxase annotation (or *MobA_MobL* for T4) was present in the majority of plasmid annotations (T5, T146, T220, T268, T271, T272, with T4 annotated as *MobA_MobL*). These annotation patterns indicate that *IncQ1/Col* replicon backgrounds and *MOB_H/MobA*-class relaxases are the principal annotated carriers of the recurrent ARG set in this dataset.

Conjugation/mobilization modules were commonly co-annotated with these replicons. Most plasmid annotations included extensive type IV secretion system (T4SS) gene lists (tfc* series and/or *virB* family members) and a type IV coupling protein (T4CP). T4CP annotations segregated into two annotation classes in the dataset: TraD (T5, T146, T220, T268, T271, T272) and FtsK-like DNA translocase (T4, T8, T140). Several strains with FtsK-like T4CP annotations (notably T8 and T140) lacked explicit IncQ1/Col replicon annotations in the provided table, although their ARG complements were similar to those of IncQ1-annotated strains.

Finally, the same plasmid/mobilome annotations that co-occurred with ARGs frequently co-localized with virulence loci (extensive *SPI-1/2*, flagellar and fimbrial gene clusters), multiple metal-resistance determinants (ars, mer, sil, pco family members) and anti-CRISPR annotations (e.g., *AcrIF7*, *AcrIIA7*, *AcrIIC1*) in the provided cargo tables. Taken together, the annotation data support a model in which a recurrent multi-gene cargo (ARGs, virulence, metal resistance, anti-CRISPR) is repeatedly associated with *IncQ1/Col-*type plasmid backgrounds and *MOB_H/MobA-*type relaxases that also encode recognizable conjugation modules (T4SS/T4CP). All conclusions are based on genome/annotation co-occurrence in the supplied tables and have not been experimentally validated for physical linkage or transferability.

## 4. Discussion

This study provided new insights into the genomic characteristics of *Salmonella* Typhimurium circulating within the pork supply chain in northeastern Thailand. Our analysis of nine representative isolates revealed a conserved genomic backbone across strains, with stable genome size, GC content, and functional profiles. Comparative genomics and COG functional annotation confirmed high conservation of core metabolic and housekeeping functions, with only minor differences across isolates, indicating that functional stability is largely maintained irrespective of seasonal origin. Nevertheless, variation in sequence types, AMR determinants, and plasmid/mobilome features highlights the ongoing diversification of this pathogen in the regional pork production system.

At the phylogenetic level, ST34 emerged as the dominant lineage, representing the majority of isolates (7/9) and encompassing both biphasic and monophasic variants. This pattern was consistent with the reports from China and Europe, where ST34 has progressively displaced ST19 as the major lineage associated with livestock and food production, particularly in swine [17,18]. Monophasic variants such as *S.* 4,(5),12:i:- are strongly linked to ST34 and have been described as globally expanding clones [19,20,21]. The co-detection of both monophasic and biphasic ST34 variants in our study suggests an ongoing evolutionary transition within the local pork supply chain, mirroring international trends. In contrast, ST19 and ST1543 appeared sporadically and carried narrower resistance repertoires, indicating more limited epidemiological relevance in this ecosystem.

The AMR gene profiles further underscored the selective advantage of ST34. Tetracycline resistance genes (*tetA/tetB*) were widely distributed, frequently co-occurring with sulfonamide (*sul2/sul3*), aminoglycoside (*aadA*, *aph(6)-Id*, *aph(3″)-Ib*), and β-lactam (*bla_TEM-1B*) determinants. Such MDR constellations closely resemble those reported in Asian pig-associated *Salmonella* isolates [22]. A large-scale genomic survey in China similarly demonstrated that ST34 carried significantly more resistance determinants than ST19, including widespread *tet*, *sul*, and *bla_TEM* genes [23]. Notably, no clear seasonal clustering of ARG repertoires was observed, suggesting that resistance gene carriage is primarily lineage-dependent rather than temporally structured. Taken together, our findings support the view that ST34 has emerged as the major MDR lineage in swine-associated *S.* Typhimurium, consolidating its role as a reservoir for resistance determinants in the food chain.

Plasmid and mobilome annotation provided additional evidence of the potential transmissibility of these ARGs. Most isolates carried *IncQ1* or *Col-*type replicons, frequently linked with *MOB_H* or *MobA* relaxases and conjugative transfer modules (T4SS/T4CP), which are characteristic of mobilizable plasmids [24]. Importantly, some plasmids also co-harbored metal resistance operons (*ars*, *mer*, *sil*, *pco*), virulence loci, and anti-CRISPR proteins. This multi-gene cargo organization suggests strong co-selection pressures: even in the absence of direct antibiotic exposure, ARGs may be stably maintained through selective advantages conferred by metal tolerance or virulence factors [25]. Such plasmid architectures reinforce the notion that AMR dissemination is not solely antibiotic-driven but may be sustained in agricultural settings by complex ecological interactions. Similarly, mobilome composition did not exhibit season-specific signatures, further supporting the predominance of lineage-associated factors in shaping accessory genome variation.

From a public health perspective, our findings carry significant implications. Pork remains a major source of animal protein in Thailand [26], and the high prevalence of MDR ST34 within the supply chain raises concerns about foodborne transmission of resistant *Salmonella* to consumers. Moreover, the resistance gene repertoire observed here mirrors those reported in human ST34 isolates in Europe and East Asia [27], suggesting that these lineages possess the genetic capacity to transcend host and geographic barriers. Given the international movement of pork products and regional trade networks, MDR ST34 strains may spread beyond local boundaries, reinforcing the need for coordinated surveillance under a One Health framework.

Nevertheless, this study has several limitations. The sample set was relatively small and restricted to a one-year period, potentially limiting the generalizability of our findings. Short-read sequencing data constrained our ability to fully resolve plasmid architectures, meaning physical linkage of ARGs and mobilome features remains to be experimentally validated. Additionally, the absence of clinical isolates precluded direct assessment of zoonotic spillover risk. Future studies integrating long-read sequencing, plasmid transfer experiments, and larger-scale epidemiological sampling will be critical to confirm the mobilization potential of the ARGs detected here and to evaluate their impact on human health.

In conclusion, our study highlights the predominance of ST34 *S*. Typhimurium in the northeastern Thailand pork supply chain, its extensive MDR gene repertoire, and its tight association with plasmid replicons and mobilization modules. These results emphasize the critical role of the pork production chain in maintaining and disseminating MDR *Salmonella* and underscore the need for enhanced genomic surveillance, risk assessment, and intervention strategies to mitigate AMR spread. By documenting the genomic and mobilome characteristics of these isolates, this work contributed to the broader understanding of one possible path in which the MDR *S*. Typhimurium evolves and persists in livestock-associated environments and provides a foundation for future One Health-based interventions.

## 5. Conclusions

This study shows that multidrug-resistant ST34 is the predominant *Salmonella* Typhimurium lineage along the pork production and retail chain in northeastern Thailand, carrying the broadest resistome and mobilizable *IncQ1/Col-*type plasmids on a largely conserved chromosomal background. To our knowledge, this is the first genomic characterization of MDR ST34 in Thailand’s pork chain, providing country-specific evidence for plasmid-mediated dissemination of resistance. Given the small number of sequenced isolates and the use of short-read assemblies, these findings should be viewed as exploratory. Future work should expand WGS surveillance to larger food, animal and human clinical collections and incorporate long-read sequencing to resolve complete plasmids and confirm their mobility in order to better inform AMR control under a One Health framework.

## Figures and Tables

**Table 1:** Sample Information.

Stains ID	Serotype	Source	Years
T4	*S.* Typhimurium	Carcass	2024
T5	Monophasci *S.* Typhimurium	Carcass	2024
T8	Monophasci *S.* Typhimurium	Carcass	2024
T140	Monophasci *S.* Typhimurium	feces	2024
T146	Monophasci *S.* Typhimurium	feces	2024
T220	*S.* Typhimurium	Pork	2024
T268	*S.* Typhimurium	Carcass	2024
T271	*S.* Typhimurium	feces	2024
T272	*S.* Typhimurium	feces	2024

**Table 2:** Genome sequencing and assembly statistics of nine representative *Salmonella* Typhimurium isolates from the pork supply chain in northeastern Thailand.

Strain ID	T4	T5	T8	T140	T146	T220	T268	T271	T272
Season	Summer	Summer	Summer	Raniy	Raniy	Raniy	Winter	Winter	Winter
Source	Carcass	Carcass	Carcass	feces	feces	Pork	Carcass	feces	feces
Reads (M)	5,737,783	5,839,821	7,328,517	7,325,203	5,226,024	42,99,303	3,433,505	4,550,328	4,657,471
Genome size (Mb)	4,761,534	4,984,354	4,844,384	4,844,384	4,991,203	4,977,876	5,000,978	4,999,702	4,949,535
GC (%)	52.14%	52.12%	52.04%	52.04%	52.15%	52.16%	52.15%	52.06%	52.16%
No. of contigs	26	58	32	32	74	71	63	61	73
N50 (kb)	531,293	280,191	594,705	594,705	224,050	223,414	236,456	224,050	223,414

**Table 3:** Transfer mechanism of plasmids associated with *Salmonella*.

Strain ID	Replicon Types	oriT/Relaxase	T4SS	T4CP
T4	*IncQ1*	*MobA_MobL*	/	*FtsK-like DNA translocase*
T5	*IncQ1*	*MOB_H*	*tfc19*; *tfc18*; *tfc22*; *tfc23*; *tfc24*; *tfc17*; *virb4*; *tfc15*; *tfc14*; *tfc13*; *tfc12*; *tfc11*; *tfc10*; *tfc9*; *tfc8*; *tfc7*; *tfc5*; *virB1*; *tfc3*; *tfc2*	*TraD*
T8	/	/	/	*FtsK-like DNA translocase*
T140	/	/	/	*FtsK-like DNA translocase*
T146	*IncQ1*	*MOB_H*	*tfc2*; *tfc3*; *tfc5*; *tfc7*; *tfc8*; *tfc9*; *tfc10*; *tfc11*; *tfc12*; *tfc13*; *tfc14*, *tfc15*; *tfc17*; *tfc18*; *tfc19*; *tfc22*; *tfc23*; *tfc24*; *virB1*; *virb4*	*TraD*
T220	*Col(pHAD28)*	*MOB_H*	*tfc2*; *tfc3*; *tfc5*; *tfc7*; *tfc8*; *tfc9*; *tfc10*; *tfc11*; *tfc12*; *tfc13*; *tfc14*; *tfc15*; *tfc17*; *tfc18*; *tfc19*; *tfc22*; *tfc23*; *tfc24*; *virB1*; *virb4*	*TraD*
T268	*IncQ1*	*MOB_H*	*tfc2*; *tfc3*; *tfc5*; *tfc7*; *tfc8*; *tfc9*; *tfc10*; *tfc11*; *tfc12*; *tfc13*; *tfc14*; *tfc15*; *tfc17*; *tfc18*; *tfc19*; *tfc22*; *tfc23*; *tfc24*; *virB1*; *virb4*	*TraD*
T271	*Col(BS512)* *IncQ1* *IncX1*	*MOB_H*	*tfc2*; *tfc3*; *tfc5*; *tfc7*; *tfc8*; *tfc9*; *tfc10*; *tfc11*; *tfc12*; *tfc13*; *tfc14*; *tfc15*; *tfc17*; *tfc18*; *tfc19*; *tfc22*; *tfc23*; *tfc24*; *virB1*; *virb4*	*TraD*
T272	*IncQ1*	*MOB_H*	*tfc2*; *tfc3*; *tfc5*; *tfc7*; *tfc8*; *tfc9*; *tfc10*; *tfc11*; *tfc12*; *tfc13*; *tfc14*; *tfc15*; *tfc17*; *tfc18*; *tfc19*; *tfc22*; *tfc23*; *tfc24*; *virB1*; *virb4*	*TraD*

**Table 4:** Motility components of plasmids associated with *Salmonella*.

Strain ID		Cargo Genes				
	**ARG**	**VF**	**Metal Resistance**	**Degradation**	**Symbiosis**	**Anti-Crispr**
T4	*aac(6′)-Iaa tet(A) aph(6)-Id aph(3″)-Ib sul2*	*SlrP*; *sopD2*; *msbA*; *nueA*; *ompA*; *pipB*; *sopB/sigD*; *csgG*; *csgF*; *csgE*; *csgD*; *csgB*; *csgA*; *csgC*; *flgB*; *flgC*; *flgE*; *flgF*; *flgG*; *flgH*; *flgI*; *flmH*; *sifA*; *spiC/ssaB*; *ssaC*; *ssaD*; *ssaE*; *sseA*; *sseB*; *sscA*; *sseC*; *sseD*; *sseE*; *sscB*; *sseF*; *sseG*; *ssaG*; *ssaH*; *ssaI*; *ssaJ*; *ssaK*; *ssaL*; *ssaM*; *ssaV*; *ssaN*; *ssaO*; *ssaP*; *ssaQ*; *ssaR*; *ssaS*; *ssaT*; *ssaU*; *sodB*; *steA*; *sifB*; *sseJ*; *steC*; *galU*; *kdsA*; *sopE2*; *flhA*; *flhB*; *cheZ*; *cheY*; *cheB*; *cheR*; *cheW*; *cheA*; *motB*; *motA*; *flhC*; *flhD*; *fliA*; *fliS*; *fliG*; *fliI*; *fliM*; *fliN*; *fliP*; *fliQ*; *fliR*; *rcsA*; *sopA*; *sopA*; *gogB*; *ugd*; *gnd*; *ddhB*; *ddhA*; *galF*; *wcaJ*; *manB*; *wcaG*; *gmd*; *wza*; *avrA*; *orgC*; *orgB/SctL*; *orgA/sctK*; *prgK*; *prgJ*; *prgI*; *prgH*; *sptP*; *sicP*; *sipA/sspA*; *sipD*; *sipC/sspC*; *sipB/sspB*; *sicA*; *spaS*; *spaR*; *spaQ*; *spaP*; *spaO/sctQ*; *invJ*; *invI*; *invC/sctN*; *invB*; *invA*; *invE*; *invG*; *invF*; *invH*; *sopD*; *AHA_3493*; *rfaE*; *wbbO*; *glf*; *entA*; *entB*; *entE*; *entC*; *fepB*; *entS*; *fepD*; *fepG*; *fepC*; *entF*; *fes*; *fepA*; *gtrA*; *gtrB*; *fimF*; *fimH*; *fimD*; *fimC*; *fimI*; *allD*; *allC*; *allB*; *allR*; *allA*; *allS*; *acrA*; *acrB*; *gmhA/lpcA*; *htpB*; *cheD*; *bcfA*; *bcfB*; *bcfC*; *bcfD*; *bcfD*; *bcfE*; *bcfF*; *bcfG*; *tufA*; *lpfE*; *lpfD*; *lpfC*; *lpfB*; *lpfA*; *rfaD*; *rfaF*; *misL*; *mgtB*; *mgtC*; *shdA*; *acrB*; *pla*; *pla*; *sseL*; *rcsB*; *lpxC*; *lpxA*; *lpxB*; *IlpA*; *iroB*; *iroC*; *iroD*; *iroN*; *pipB2*; *mig-14*; *luxS*; *algU*; *sinH*; *ratB*; *shdA*; *acrB*; *acrA*; *tufA*; *tufA*	*modE*; *modA*; *modB*; *modC*; *mntR*; *kdeA*; *nfsA*; *comR/ycfQ*; *bhsA/ycfR/comC*; *phoB*; *zinT/yodA*; *kpnO*; *kpnE*; *kpnF*; *kpnO*; *kpnO*; *kmrA*; *dsbB*; *znuA/yebL*; *znuC/yebM*; *znuB/yebI*; *ruvB*; *cutC*; *kpnO*; *arsA*; *mdtA*; *mdtB*; *mdtC*; *rcnB/yohN*; *yfeC*; *rcnR/yohL*; *rcnA/yohM*; *dsbC*; *zupT/ygiE*; *yqjH*; *zitB/ybgR*; *corC*; *cutE/lnt*; *cueR/ybbI*; *copA*; *acrD/yffA*; *golS*; *golT*; *zur/yjbK*; *actP/yjcG*; *cutA*; *mgtA*; *zntA/yhhO*; *nikR*; *cueP*; *acrD*; *mntH/yfeP*; *pmrG*; *kpnO*; *corD*; *cuiD*; *cutF/nlpE*; *oxyRkp*; *fieF/yiip*; *cpxR*; *cpxA*; *dsbA*; *corA*; *pstB*; *pstA*; *pstC*; *pstS*; *acrD/yffA*; *zraR/hydH*; *zraP*; *zntR/yhdM*	*bhpD*; *dcl*	*NoeL*	*AcrIF7*; *AcrIIA7*; *AcrIIA7*; *AcrIIC1*
T5	*aac(6′)-Iaa*; *tet(B)*; *aph(6)-Id*; *aph(3″)-Ib*; *sul2*; *blaTEM-1B*	*sodCI*; *sseI/srfH*; *ompA*; *pipB*; *sopB/sigD*; *csgG*; *csgF*; *csgE*; *csgD*; *csgB*; *csgA*; *csgC*; *flgB*; *flgC*; *flgE*; *flgF*; *flgG*; *flgH*; *flgI*; *flmH*; *sifA*; *spiC/ssaB*; *ssaC*; *ssaD*; *ssaE*; *sseA*; *sseB*; *sscA*; *sseC*; *sseD*; *sseE*; *sscB*; *sseF*; *sseG*; *ssaG*; *ssaH*; *ssaI*; *ssaJ*; *ssaK*; *ssaL*; *ssaM*; *ssaV*; *ssaN*; *ssaO*; *ssaP*; *ssaQ*; *ssaR*; *ssaS*; *ssaT*; *ssaU*; *sodB*; *steA*; *sifB*; *sseJ*; *steC*; *galU*; *kdsA*; *sopE2*; *flhA*; *flhB*; *cheZ*; *cheY*; *cheB*; *cheR*; *cheW*; *cheA*; *motB*; *motA*; *flhC*; *flhD*; *fliA*; *iroB*; *iroC*; *iroD*; *iroN*; *pipB2*; *mig-14*; *luxS*; *avrA*; *orgC*; *orgB/SctL*; *orgA/sctK*; *prgK*; *prgJ*; *prgI*; *prgH*; *sptP*; *sicP*; *sipA/sspA*; *sipD*; *sipC/sspC*; *sipB/sspB*; *sicA*; *spaS*; *spaR*; *spaQ*; *spaP*; *spaO/sctQ*; *invJ*; *invI*; *invC/sctN*; *invB*; *invA*; *invE*; *invG*; *invF*; *invH*; *sopD*; *AHA_3493*; *rfaE*; *mgtC*; *mgtB*; *misL*; *rfaF*; *rfaD*; *lpfA*; *lpfB*; *lpfC*; *lpfD*; *lpfE*; *acrB*; *acrA*; *allS*; *allA*; *allR*; *allB*; *allC*; *allD*; *fimI*; *fimC*; *fimD*; *fimH*; *fimF*; *gtrB*; *gtrA*; *fepA*; *fes*; *entF*; *fepC*; *fepG*; *fepD*; *entS*; *fepB*; *entC*; *entE*; *entB*; *entA*; *glf*; *wbbO*; *bcfG*; *bcfF*; *bcfE*; *bcfD*; *bcfC*; *bcfB*; *bcfA*; *cheD*; *gtrA*; *gtrB*; *gmhA/lpcA*; *hsiC1/vipB*; *hsiB1/vipA*; *htpB*; *sseK2*; *wza*; *gmd*; *wcaG*; *manB*; *wcaJ*; *galF*; *ddhA*; *ddhB*; *gnd*; *ugd*; *sopA*; *IlpA*; *lpxB*; *lpxA*; *lpxC*; *rcsB*; *sseL*; *shdA*; *ratB*; *sinH*; *gogB*; *gtrB*; *sopD2*; *msbA*; *nueA*; *acrB*; *shdA*; *pla*; *fliS*; *fliG*; *fliI*; *fliM*; *fliN*; *fliP*; *fliQ*; *fliR*; *rcsA*; *acrB*; *acrA*; *slrP*; *tufA*; *tufA*; *sseK1*; *algU*; *sspH2*; *tufA*	*comR/ycfQ*; *bhsA/ycfR/comC*; *phoB*; *zinT/yodA*; *kpnO*; *kpnE*; *kpnF*; *kpnO*; *kpnO*; *kmrA*; *dsbB*; *znuA/yebL*; *znuC/yebM*; *znuB/yebI*; *ruvB*; *cutC*; *yfeC*; *rcnR/yohL*; *rcnA/yohM*; *dsbC*; *zupT/ygiE*; *yqjH*; *cueP*; *nikR*; *zntA/yhhO*; *acrD/yffA*; *copA*; *cueR/ybbI*; *cusS*; *cutE/lnt*; *corC*; *zitB/ybgR*; *mgtA*; *golS*; *golT*; *cutA*; *pcoE*; *pcoS*; *pcoR*; *pcoD*; *pcoC*; *pcoB*; *pcoA*; *silP*; *silA*; *silB*; *silF*; *silC*; *silR*; *silS*; *silE*; *arsC*; *arsB*; *arsA*; *arsD*; *arsR*; *actP/yjcG*; *rcnB/yohN*; *mdtC*; *mdtB*; *mdtA*; *cutF/nlpE*; *cuiD*; *corD*; *oxyRkp*; *fieF/yiip*; *cpxR*; *cpxA*; *dsbA*; *kpnO*; *pmrG*; *mntR*; *kdeA*; *nfsA*; *corA*; *pstS*; *pstC*; *pstA*; *pstB*; *zur/yjbK*; *acrD*; *mntH/yfeP*; *kpnO*; *acrD/yffA*; *modC*; *modB*; *modA*; *modE*; *zraP*; *zraR/hydH*; *zntR/yhdM*; *merR*; *merT*; *merP*; *merA*; *merD*; *merE*	*bhpD*; *dcl*	*NoeL*	*AcrIF7*; *AcrIIA7*; *AcrIIA7*; *AcrIIC1*
T8	*aac(6′)-Iaa*; *tet(A)*; *aadA2*; *cmlA1*; *ant(3″)-Ia*; *sul3*; *aac(3)-IVa*; *blaTEM-1B*	*glf*; *wbbO*; *slrP*; *sopD2*; *msbA*; *nueA*; *ompA*; *pipB*; *sopB/sigD*; *csgG*; *csgF*; *csgE*; *csgD*; *csgB*; *csgA*; *csgC*; *flgB*; *flgC*; *flgE*; *flgF*; *flgG*; *flgH*; *flgI*; *flmH*; *sifA*; *spiC/ssaB*; *ssaC*; *ssaD*; *ssaE*; *sseA*; *sseB*; *sscA*; *sseC*; *sseD*; *sseE*; *sscB*; *sseF*; *sseG*; *ssaG*; *ssaH*; *ssaI*; *ssaJ*; *ssaK*; *ssaL*; *ssaM*; *ssaV*; *ssaN*; *ssaO*; *ssaP*; *ssaQ*; *ssaR*; *ssaS*; *ssaT*; *ssaU*; *sodB*; *steA*; *sifB*; *sseJ*; *steC*; *galU*; *IlpA*; *lpxB*; *lpxA*; *lpxD*; *lpxC*; *bcfG*; *bcfF*; *bcfE*; *bcfD*; *bcfC*; *bcfB*; *bcfA*; *cheD*; *htpB*; *gtrB*; *avrA*; *orgC*; *orgB/SctL*; *orgA/sctK*; *prgK*; *prgJ*; *prgI*; *prgH*; *sptP*; *sicP*; *sipA/sspA*; *sipD*; *sipC/sspC*; *sipB/sspB*; *sicA*; *spaS*; *spaR*; *spaQ*; *spaP*; *spaO/sctQ*; *invJ*; *invI*; *invC/sctN*; *invB*; *invA*; *invE*; *invG*; *invF*; *invH*; *sopD*; *AHA_3493*; *rfaE*; *acrA*; *acrB*; *mgtC*; *mgtB*; *misL*; *rfaF*; *rfaD*; *lpfA*; *lpfB*; *lpfC*; *lpfD*; *lpfE*; *rcsB*; *sseL*; *acrB*; *shdA*; *ratB*; *sinH*; *algU*; *hsiB1/vipA*; *hsiC1/vipB*; *gmhA/lpcA*; *acrB*; *acrA*; *allS*; *allA*; *allR*; *allB*; *allC*; *allD*; *fimI*; *fimC*; *fimD*; *fimH*; *fimF*; *gtrB*; *gtrA*; *fepA*; *fes*; *entF*; *fepC*; *fepG*; *fepD*; *entS*; *fepB*; *entC*; *entE*; *entB*; *entA*; *kdsA*; *sopE2*; *sspH2*; *flhA*; *flhB*; *cheZ*; *cheY*; *cheB*; *cheR*; *cheW*; *cheA*; *motB*; *motA*; *flhC*; *flhD*; *fliA*; *fliC*; *sseK2*; *wza*; *gmd*; *wcaG*; *manB*; *manB*; *gnd*; *ugd*; *sopA*; *rcsA*; *fliR*; *fliQ*; *fliP*; *fliN*; *fliM*; *fliI*; *fliG*; *fliF*; *fliS*; *wbtL*; *fliC*; *iroB*; *iroC*; *iroD*; *iroN*; *pipB2*; *mig-14*; *luxS*; *tufA*; *sseK1*; *manB*; *wcaG*; *gmd*; *galF*; *wcaJ*; *manB*; *tufA*	*cutE/lnt*; *corC*; *zitB/ybgR*; *modE*; *modA*; *modB*; *modC*; *mntR*; *kdeA*; *nfsA*; *comR/ycfQ*; *bhsA/ycfR/comC*; *phoB*; *zinT/yodA*; *kpnO*; *kpnE*; *kpnF*; *kpnO*; *kpnO*; *kmrA*; *cutF/nlpE*; *cuiD*; *corD*; *mgtA*; *cutA*; *actP/yjcG*; *zur/yjbK*; *yfeC*; *rcnR/yohL*; *rcnA/yohM*; *dsbC*; *zupT/ygiE*; *yqjH*; *acrD/yffA*; *cueP*; *nikR*; *zntA/yhhO*; *kpnO*; *pmrG*; *mntH/yfeP*; *acrD*; *golT*; *golS*; *acrD/yffA*; *copA*; *cueR/ybbI*; *cusS*; *dsbB*; *znuA/yebL*; *znuC/yebM*; *znuB/yebI*; *ruvB*; *cutC*; *rcnB/yohN*; *mdtC*; *mdtB*; *mdtA*; *oxyRkp*; *fieF/yiip*; *cpxR*; *cpxA*; *dsbA*; *arsA*; *kpnO*; *corA*; *pstS*; *pstC*; *pstA*; *pstB*; *zraP*; *zraR/hydH*; *zntR/yhdM*; *silE*; *silS*; *silR*; *silC*; *silF*; *silB*; *silA*; *silP*; *merR*; *merT*; *merP*; *merA*; *merD*; *merE*	*dcl*; *bhpD*	*NoeL*; *NoeL*	*AcrIF7*; *AcrIIA7*; *AcrIIA7*; *AcrIIC1*
T140	*aac(6′)-Iaa*; *tet(A)*; *aadA2*; *cmlA1*; *ant(3″)-Ia*; *sul3*; *aac(3)-IVa*; *blaTEM-1B*	*glf*; *wbbO*; *slrP*; *sopD2*; *msbA*; *nueA*; *ompA*; *pipB*; *sopB/sigD*; *csgG*; *csgF*; *csgE*; *csgD*; *csgB*; *csgA*; *csgC*; *flgB*; *flgC*; *flgE*; *flgF*; *flgG*; *flgH*; *flgI*; *flmH*; *sifA*; *spiC/ssaB*; *ssaC*; *ssaD*; *ssaE*; *sseA*; *sseB*; *sscA*; *sseC*; *sseD*; *sseE*; *sscB*; *sseF*; *sseG*; *ssaG*; *ssaH*; *ssaI*; *ssaJ*; *ssaK*; *ssaL*; *ssaM*; *ssaV*; *ssaN*; *ssaO*; *ssaP*; *ssaQ*; *ssaR*; *ssaS*; *ssaT*; *ssaU*; *sodB*; *steA*; *sifB*; *sseJ*; *steC*; *galU*; *IlpA*; *lpxB*; *lpxA*; *lpxD*; *lpxC*; *bcfG*; *bcfF*; *bcfE*; *bcfD*; *bcfC*; *bcfB*; *bcfA*; *cheD*; *htpB*; *gtrB*; *avrA*; *orgC*; *orgB/SctL*; *orgA/sctK*; *prgK*; *prgJ*; *prgI*; *prgH*; *sptP*; *sicP*; *sipA/sspA*; *sipD*; *sipC/sspC*; *sipB/sspB*; *sicA*; *spaS*; *spaR*; *spaQ*; *spaP*; *spaO/sctQ*; *invJ*; *invI*; *invC/sctN*; *invB*; *invA*; *invE*; *invG*; *invF*; *invH*; *sopD*; *AHA_3493*; *rfaE*; *acrA*; *acrB*; *mgtC*; *mgtB*; *misL*; *rfaF*; *rfaD*; *lpfA*; *lpfB*; *lpfC*; *lpfD*; *lpfE*; *rcsB*; *sseL*; *acrB*; *shdA*; *ratB*; *sinH*; *algU*; *hsiB1/vipA*; *hsiC1/vipB*; *gmhA/lpcA*; *acrB*; *acrA*; *allS*; *allA*; *allR*; *allB*; *allC*; *allD*; *fimI*; *fimC*; *fimD*; *fimH*; *fimF*; *gtrB*; *gtrA*; *fepA*; *fes*; *entF*; *fepC*; *fepG*; *fepD*; *entS*; *fepB*; *entC*; *entE*; *entB*; *entA*; *kdsA*; *sopE2*; *sspH2*; *flhA*; *flhB*; *cheZ*; *cheY*; *cheB*; *cheR*; *cheW*; *cheA*; *motB*; *motA*; *flhC*; *flhD*; *fliA*; *fliC*; *sseK2*; *wza*; *gmd*; *wcaG*; *manB*; *manB*; *gnd*; *ugd*; *sopA*; *rcsA*; *fliR*; *fliQ*; *fliP*; *fliN*; *fliM*; *fliI*; *fliG*; *fliF*; *fliS*; *wbtL*; *fliC*; *iroB*; *iroC*; *iroD*; *iroN*; *pipB2*; *mig-14*; *luxS*; *tufA*; *sseK1*; *manB*; *wcaG*; *gmd*; *galF*; *wcaJ*; *manB*; *tufA*	*cutE/lnt*; *corC*; *zitB/ybgR*; *modE*; *modA*; *modB*; *modC*; *mntR*; *kdeA*; *nfsA*; *comR/ycfQ*; *bhsA/ycfR/comC*; *phoB*; *zinT/yodA*; *kpnO*; *kpnE*; *kpnF*; *kpnO*; *kpnO*; *kmrA*; *cutF/nlpE*; *cuiD*; *corD*; *mgtA*; *cutA*; *actP/yjcG*; *zur/yjbK*; *yfeC*; *rcnR/yohL*; *rcnA/yohM*; *dsbC*; *zupT/ygiE*; *yqjH*; *acrD/yffA*; *cueP*; *nikR*; *zntA/yhhO*; *kpnO*; *pmrG*; *mntH/yfeP*; *acrD*; *golT*; *golS*; *acrD/yffA*; *copA*; *cueR/ybbI*; *cusS*; *dsbB*; *znuA/yebL*; *znuC/yebM*; *znuB/yebI*; *ruvB*; *cutC*; *rcnB/yohN*; *mdtC*; *mdtB*; *mdtA*; *oxyRkp*; *fieF/yiip*; *cpxR*; *cpxA*; *dsbA*; *arsA*; *kpnO*; *corA*; *pstS*; *pstC*; *pstA*; *pstB*; *zraP*; *zraR/hydH*; *zntR/yhdM*; *silE*; *silS*; *silR*; *silC*; *silF*; *silB*; *silA*; *silP*; *merR*; *merT*; *merP*; *merA*; *merD*; *merE*	*dcl*; *bhpD*	*NoeL*; *NoeL*	*AcrIF7*; *AcrIIA7*; *AcrIIA7*; *AcrIIC1*
T146	*aac(6′)-Iaa*; *aph(6)-Id*; *aph(3″)-Ib*; *sul2*; *blaTEM-1B*	*kdsA*; *galU*; *steC*; *sseJ*; *sifB*; *steA*; *sodB*; *ssaU*; *ssaT*; *ssaS*; *ssaR*; *ssaQ*; *ssaP*; *ssaO*; *ssaN*; *ssaV*; *ssaM*; *ssaL*; *ssaK*; *ssaJ*; *ssaI*; *ssaH*; *ssaG*; *sseG*; *sseF*; *sscB*; *sseE*; *sseD*; *sseC*; *sscA*; *sseB*; *sseA*; *ssaE*; *ssaD*; *ssaC*; *spiC/ssaB*; *sifA*; *flmH*; *flgI*; *flgH*; *flgG*; *flgF*; *flgE*; *flgC*; *flgB*; *csgC*; *csgA*; *csgB*; *csgD*; *csgE*; *csgF*; *csgG*; *sopB/sigD*; *pipB*; *ompA*; *sseI/srfH*; *sopD*; *AHA_3493*; *rfaE*; *lpfE*; *lpfD*; *lpfC*; *lpfB*; *lpfA*; *rfaD*; *rfaF*; *misL*; *wbbO*; *glf*; *entA*; *entB*; *entE*; *entC*; *fepB*; *entS*; *fepD*; *fepG*; *fepC*; *entF*; *fes*; *fepA*; *gtrA*; *gtrB*; *fimF*; *fimH*; *fimD*; *fimC*; *fimI*; *allD*; *allC*; *allB*; *allR*; *allA*; *allS*; *acrA*; *acrB*; *cheD*; *bcfA*; *bcfB*; *bcfC*; *bcfD*; *bcfE*; *bcfF*; *bcfG*; *htpB*; *IlpA*; *lpxB*; *lpxA*; *lpxC*; *sseK2*; *wza*; *gmd*; *wcaG*; *manB*; *wcaJ*; *galF*; *ddhA*; *ddhB*; *gnd*; *ugd*; *sopA*; *gtrA*; *gtrB*; *acrB*; *shdA*; *ratB*; *sinH*; *gogB*; *sseL*; *rcsB*; *luxS*; *avrA*; *orgC*; *orgB/SctL*; *orgA/sctK*; *prgK*; *prgJ*; *prgI*; *prgH*; *sptP*; *sicP*; *sipA/sspA*; *sipD*; *sipC/sspC*; *sipB/sspB*; *sicA*; *spaS*; *spaR*; *spaQ*; *spaP*; *spaO/sctQ*; *invJ*; *invI*; *invC/sctN*; *invB*; *invA*; *invE*; *invG*; *invF*; *invH*; *sopE2*; *flhA*; *flhB*; *cheZ*; *cheY*; *cheB*; *cheR*; *cheW*; *cheA*; *motB*; *motA*; *flhC*; *flhD*; *fliA*; *gtrB*; *sopD2*; *msbA*; *nueA*; *pla*; *fliS*; *fliG*; *fliI*; *fliM*; *fliN*; *fliP*; *fliQ*; *fliR*; *rcsA*; *hsiC1/vipB*; *hsiB1/vipA*; *mgtC*; *mgtB*; *mig-14*; *pipB2*; *iroN*; *iroD*; *iroC*; *iroB*; *acrA*; *acrB*; *slrP*; *tufA*; *sseK1*; *gmhA/lpcA*; *algU*; *sodCI*; *sspH2*; *tufA*	*dsbB*; *kmrA*; *kpnO*; *kpnO*; *kpnF*; *kpnE*; *kpnO*; *zinT/yodA*; *phoB*; *bhsA/ycfR/comC*; *comR/ycfQ*; *rcnR/yohL*; *rcnA/yohM*; *dsbC*; *zupT/ygiE*; *yqjH*; *zntA/yhhO*; *nikR*; *cueP*; *zitB/ybgR*; *corC*; *cutE/lnt*; *cusS*; *cueR/ybbI*; *copA*; *acrD/yffA*; *mgtA*; *actP/yjcG*; *arsR*; *arsD*; *arsA*; *arsB*; *arsC*; *silE*; *silS*; *silR*; *silC*; *silF*; *silB*; *silA*; *silP*; *pcoA*; *pcoB*; *pcoC*; *pcoD*; *pcoR*; *pcoS*; *pcoE*; *cutA*; *cutF/nlpE*; *cuiD*; *corD*; *rcnB/yohN*; *mdtC*; *mdtB*; *mdtA*; *golS*; *golT*; *acrD*; *dsbA*; *cpxA*; *cpxR*; *fieF/yiip*; *oxyRkp*; *pmrG*; *kpnO*; *yfeC*; *znuA/yebL*; *znuC/yebM*; *znuB/yebI*; *ruvB*; *cutC*; *corA*; *pstS*; *pstC*; *pstA*; *pstB*; *zur/yjbK*; *nfsA*; *kdeA*; *mntH/yfeP*; *kpnO*; *acrD/yffA*; *modE*; *modA*; *modB*; *modC*; *zraP*; *zraR/hydH*; *mntR*; *zntR/yhdM*; *merR*; *merT*; *merP*; *merA*; *merD*; *merE*	*bhpD*; *dcl*	*NoeL*	*AcrIF7*; *AcrIIA7*; *AcrIIC1*; *AcrIIA7*
T220	*aac(6′)-Iaa*; *tet(B)*; *sul2*; *aph(3″)-Ib*; *aph(6)-Id*; *qnrB19*; *blaTEM-1B*	*aac(6′)-Iaa*; *tet(B)*; *sul2*; *aph(3″)-Ib*; *aph(6)-Id*; *qnrB19*; *blaTEM-1B*	*dsbB*; *kmrA*; *kpnO*; *kpnO*; *kpnF*; *kpnE*; *kpnO*; *zinT/yodA*; *phoB*; *bhsA/ycfR/comC*; *comR/ycfQ*; *zntA/yhhO*; *nikR*; *cueP*; *zitB/ybgR*; *corC*; *cutE/lnt*; *cusS*; *cueR/ybbI*; *copA*; *acrD/yffA*; *actP/yjcG*; *arsR*; *arsD*; *arsA*; *arsB*; *arsC*; *silE*; *silS*; *silR*; *silC*; *silF*; *silB*; *silA*; *silP*; *pcoA*; *pcoB*; *pcoC*; *pcoD*; *pcoR*; *pcoS*; *pcoE*; *cutA*; *mgtA*; *yqjH*; *zupT/ygiE*; *rcnR/yohL*; *rcnA/yohM*; *dsbC*; *cutF/nlpE*; *cuiD*; *corD*; *rcnB/yohN*; *mdtC*; *mdtB*; *mdtA*; *golS*; *golT*; *acrD*; *oxyRkp*; *fieF/yiip*; *cpxR*; *cpxA*; *dsbA*; *znuA/yebL*; *znuC/yebM*; *znuB/yebI*; *ruvB*; *cutC*; *kpnO*; *pmrG*; *yfeC*; *corA*; *pstB*; *pstA*; *pstC*; *pstS*; *zur/yjbK*; *kdeA*; *nfsA*; *mntH/yfeP*; *kpnO*; *acrD/yffA*; *modC*; *modB*; *modA*; *modE*; *zraP*; *zraR/hydH*; *mntR*; *zntR/yhdM*; *merR*; *merT*; *merP*; *merA*; *merD*; *merE*	*bhpD*; *dcl*	*NoeL*	*AcrIF7*; *AcrIIC1*; *AcrIIA7*
T268	*aac(6′)-Iaa*; *aph(6)-Id*; *aph(3″)-Ib*; *sul2*; *blaTEM-1B*	*csgG*; *csgF*; *csgE*; *csgD*; *csgB*; *csgA*; *csgC*; *flgB*; *flgC*; *flgE*; *flgF*; *flgG*; *flgH*; *flgI*; *flmH*; *sifA*; *spiC/ssaB*; *ssaC*; *ssaD*; *ssaE*; *sseA*; *sseB*; *sscA*; *sseC*; *sseD*; *sseE*; *sscB*; *sseF*; *sseG*; *ssaG*; *ssaH*; *ssaI*; *ssaJ*; *ssaK*; *ssaL*; *ssaM*; *ssaV*; *ssaN*; *ssaO*; *ssaP*; *ssaQ*; *ssaR*; *ssaS*; *ssaT*; *ssaU*; *sodB*; *steA*; *sifB*; *sseJ*; *steC*; *galU*; *kdsA*; *rfaE*; *AHA_3493*; *sopD*; *invH*; *invF*; *invG*; *invE*; *invA*; *invB*; *invC/sctN*; *invI*; *invJ*; *spaO/sctQ*; *spaP*; *spaQ*; *spaR*; *spaS*; *sicA*; *sipB/sspB*; *sipC/sspC*; *sipD*; *sipA/sspA*; *sicP*; *sptP*; *prgH*; *prgI*; *prgJ*; *prgK*; *orgA/sctK*; *orgB/SctL*; *orgC*; *avrA*; *luxS*; *lpfE*; *lpfD*; *lpfC*; *lpfB*; *lpfA*; *rfaD*; *rfaF*; *misL*; *mgtB*; *mgtC*; *wbbO*; *glf*; *entA*; *entB*; *entE*; *entC*; *fepB*; *entS*; *fepD*; *fepG*; *fepC*; *entF*; *fes*; *fepA*; *gtrA*; *gtrB*; *fimF*; *fimH*; *fimD*; *fimC*; *fimI*; *allD*; *allC*; *allB*; *allR*; *allA*; *allS*; *acrA*; *acrB*; *bcfG*; *bcfF*; *bcfE*; *bcfD*; *bcfC*; *bcfB*; *bcfA*; *cheD*; *sseK2*; *wza*; *gmd*; *wcaG*; *manB*; *wcaJ*; *galF*; *ddhA*; *ddhB*; *gnd*; *ugd*; *sopA*; *htpB*; *lpxC*; *lpxA*; *lpxB*; *IlpA*; *gtrA*; *gtrB*; *rcsB*; *sseL*; *shdA*; *ratB*; *sinH*; *gogB*; *fliA*; *flhD*; *flhC*; *motA*; *motB*; *cheA*; *cheW*; *cheR*; *cheB*; *cheY*; *cheZ*; *flhB*; *flhA*; *sopE2*; *sodCI*; *sseI/srfH*; *ompA*; *pipB*; *sopB/sigD*; *acrB*; *shdA*; *gmhA/lpcA*; *hsiC1/vipB*; *hsiB1/vipA*; *gtrB*; *nueA*; *msbA*; *sopD2*; *pla*; *tufA*; *rcsA*; *fliR*; *fliQ*; *fliP*; *fliN*; *fliM*; *fliI*; *fliG*; *fliS*; *iroB*; *iroC*; *iroD*; *iroN*; *pipB2*; *mig-14*; *acrA*; *acrB*; *slrP*; *sseK1*; *tufA*; *algU*; *sspH2*	*comR/ycfQ*; *bhsA/ycfR/comC*; *phoB*; *zinT/yodA*; *kpnO*; *kpnE*; *kpnF*; *kpnO*; *kpnO*; *kmrA*; *dsbB*; *yqjH*; *zupT/ygiE*; *dsbC*; *rcnA/yohM*; *rcnR/yohL*; *yfeC*; *zntA/yhhO*; *nikR*; *cueP*; *zitB/ybgR*; *corC*; *cutE/lnt*; *cusS*; *cueR/ybbI*; *copA*; *acrD/yffA*; *mgtA*; *rcnB/yohN*; *mdtC*; *mdtB*; *mdtA*; *cutA*; *pcoE*; *pcoS*; *pcoR*; *pcoD*; *pcoC*; *pcoB*; *pcoA*; *silP*; *silA*; *silB*; *silF*; *silC*; *silR*; *silS*; *silE*; *arsC*; *arsB*; *arsA*; *arsD*; *arsR*; *actP/yjcG*; *corD*; *cuiD*; *cutF/nlpE*; *golS*; *golT*; *oxyRkp*; *fieF/yiip*; *cpxR*; *cpxA*; *dsbA*; *kpnO*; *pmrG*; *mntR*; *kdeA*; *nfsA*; *cutC*; *ruvB*; *znuB/yebI*; *znuC/yebM*; *znuA/yebL*; *corA*; *acrD*; *pstB*; *pstA*; *pstC*; *pstS*; *zur/yjbK*; *mntH/yfeP*; *zntR/yhdM*; *kpnO*; *acrD/yffA*; *modC*; *modB*; *modA*; *modE*; *zraR/hydH*; *zraP*; *merE*; *merD*; *merA*; *merP*; *merT*; *merR*	*dcl*; *bhpD*	*NoeL*	*AcrIF7*; *AcrIIA7*; *AcrIIA7*; *AcrIIC1*
T271	*aac(6′)-Iaa*; *tet(B)*; *aph(6)-Id*; *aph(3″)-Ib*; *sul2*; *blaTEM-1B*	*sifA*; *spiC/ssaB*; *ssaC*; *ssaD*; *ssaE*; *sseA*; *sseB*; *sscA*; *sseC*; *sseD*; *sseE*; *sscB*; *sseF*; *sseG*; *ssaG*; *ssaH*; *ssaI*; *ssaJ*; *ssaK*; *ssaL*; *ssaM*; *ssaV*; *ssaN*; *ssaO*; *ssaP*; *ssaQ*; *ssaR*; *ssaS*; *ssaT*; *ssaU*; *sodB*; *steA*; *sifB*; *sseJ*; *steC*; *galU*; *kdsA*; *sopE2*; *flhA*; *flhB*; *cheZ*; *cheY*; *cheB*; *cheR*; *cheW*; *cheA*; *motB*; *motA*; *flhC*; *flhD*; *fliA*; *AHA_3493*; *sopD*; *invH*; *invF*; *invG*; *invE*; *invA*; *invB*; *invC/sctN*; *invI*; *invJ*; *spaO/sctQ*; *spaP*; *spaQ*; *spaR*; *spaS*; *sicA*; *sipB/sspB*; *sipC/sspC*; *sipD*; *sipA/sspA*; *sicP*; *sptP*; *prgH*; *prgI*; *prgJ*; *prgK*; *orgA/sctK*; *orgB/SctL*; *orgC*; *avrA*; *luxS*; *mig-14*; *pipB2*; *iroN*; *iroD*; *iroC*; *iroB*; *misL*; *rfaF*; *rfaD*; *lpfA*; *lpfB*; *lpfC*; *lpfD*; *lpfE*; *cheD*; *bcfA*; *bcfB*; *bcfC*; *bcfD*; *bcfE*; *bcfF*; *bcfG*; *htpB*; *flmH*; *flgI*; *flgH*; *flgG*; *flgF*; *flgE*; *flgC*; *flgB*; *csgC*; *csgA*; *csgB*; *csgD*; *csgE*; *csgF*; *csgG*; *sopB/sigD*; *pipB*; *ompA*; *sseI/srfH*; *sodCI*; *nueA*; *msbA*; *sopD2*; *lpxC*; *lpxA*; *lpxB*; *IlpA*; *sopA*; *ugd*; *gnd*; *ddhB*; *ddhA*; *galF*; *wcaJ*; *manB*; *wcaG*; *gmd*; *wza*; *sseK2*; *wbbO*; *glf*; *entA*; *entB*; *entE*; *entC*; *fepB*; *entS*; *fepD*; *fepG*; *fepC*; *entF*; *fes*; *fepA*; *acrB*; *shdA*; *ratB*; *sinH*; *algU*; *sseL*; *rcsB*; *rfaE*; *gtrA*; *gtrB*; *fimF*; *fimH*; *fimD*; *fimC*; *fimI*; *allD*; *allC*; *allB*; *allR*; *allA*; *allS*; *acrA*; *acrB*; *gtrB*; *pla*; *fliS*; *fliG*; *fliI*; *fliM*; *fliN*; *fliP*; *fliQ*; *fliR*; *rcsA*; *hsiC1/vipB*; *hsiB1/vipA*; *acrB*; *acrA*; *slrP*; *sseK1*; *gmhA/lpcA*; *mgtC*; *mgtB*; *sspH2*; *tufA*	*phoB*; *zinT/yodA*; *kpnO*; *kpnE*; *kpnF*; *kpnO*; *kpnO*; *kmrA*; *dsbB*; *znuA/yebL*; *znuC/yebM*; *znuB/yebI*; *ruvB*; *cutC*; *dsbC*; *rcnA/yohM*; *rcnR/yohL*; *yfeC*; *cueP*; *nikR*; *zntA/yhhO*; *mgtA*; *cutA*; *pcoE*; *pcoS*; *pcoR*; *pcoD*; *pcoC*; *pcoB*; *pcoA*; *silP*; *silA*; *silB*; *silF*; *silC*; *silR*; *silS*; *silE*; *arsC*; *arsB*; *arsA*; *arsD*; *arsR*; *actP/yjcG*; *bhsA/ycfR/comC*; *comR/ycfQ*; *corD*; *cuiD*; *cutF/nlpE*; *mdtA*; *mdtB*; *mdtC*; *rcnB/yohN*; *zitB/ybgR*; *corC*; *cutE/lnt*; *cusS*; *acrD*; *golS*; *golT*; *dsbA*; *cpxA*; *cpxR*; *fieF/yiip*; *oxyRkp*; *pmrG*; *kpnO*; *yqjH*; *zupT/ygiE*; *cueR/ybbI*; *copA*; *acrD/yffA*; *corA*; *pstB*; *pstA*; *pstC*; *pstS*; *zur/yjbK*; *nfsA*; *kdeA*; *mntH/yfeP*; *kpnO*; *acrD/yffA*; *modC*; *modB*; *modA*; *modE*; *zraP*; *zraR/hydH*; *mntR*; *zntR/yhdM*; *merE*; *merD*; *merA*; *merP*; *merT*; *merR*	*bhpD*; *dcl*	*NoeL*	*AcrIIA7*; *AcrIF7*; *AcrIIC1*
T272	*aac(6′)-Iaa*; *tet(B)*; *sul2*; *aph(3″)-Ib*; *aph(6)-Id*; *blaTEM-1B*	*gogB*; *sinH*; *ratB*; *shdA*; *acrB*; *csgG*; *csgF*; *csgE*; *csgD*; *csgB*; *csgA*; *csgC*; *flgB*; *flgC*; *flgE*; *flgF*; *flgG*; *flgH*; *flgI*; *flmH*; *sifA*; *spiC/ssaB*; *ssaC*; *ssaD*; *ssaE*; *sseA*; *sseB*; *sscA*; *sseC*; *sseD*; *sseE*; *sscB*; *sseF*; *sseG*; *ssaG*; *ssaH*; *ssaI*; *ssaJ*; *ssaK*; *ssaL*; *ssaM*; *ssaV*; *ssaN*; *ssaO*; *ssaP*; *ssaQ*; *ssaR*; *ssaS*; *ssaT*; *ssaU*; *sodB*; *steA*; *sifB*; *sseJ*; *steC*; *galU*; *kdsA*; *sopE2*; *flhA*; *flhB*; *cheZ*; *cheY*; *cheB*; *cheR*; *cheW*; *cheA*; *motB*; *motA*; *flhC*; *flhD*; *fliA*; *iroB*; *iroC*; *iroD*; *iroN*; *pipB2*; *mig-14*; *luxS*; *avrA*; *orgC*; *orgB/SctL*; *orgA/sctK*; *prgK*; *prgJ*; *prgI*; *prgH*; *sptP*; *sicP*; *sipA/sspA*; *sipD*; *sipC/sspC*; *sipB/sspB*; *sicA*; *spaS*; *spaR*; *spaQ*; *spaP*; *spaO/sctQ*; *invJ*; *invI*; *invC/sctN*; *invB*; *invA*; *invE*; *invG*; *invF*; *invH*; *sopD*; *AHA_3493*; *lpfE*; *lpfD*; *lpfC*; *lpfB*; *lpfA*; *rfaD*; *rfaF*; *misL*; *cheD*; *bcfA*; *bcfB*; *bcfC*; *bcfD*; *bcfE*; *bcfF*; *bcfG*; *htpB*; *lpxC*; *lpxA*; *lpxB*; *IlpA*; *sopA*; *ugd*; *gnd*; *ddhB*; *ddhA*; *galF*; *wcaJ*; *manB*; *wcaG*; *gmd*; *wza*; *sseK2*; *wbbO*; *glf*; *entA*; *entB*; *entE*; *entC*; *fepB*; *entS*; *fepD*; *fepG*; *fepC*; *entF*; *fes*; *fepA*; *rfaE*; *gmhA/lpcA*; *sseL*; *rcsB*; *gtrA*; *gtrB*; *fimF*; *fimH*; *fimD*; *fimC*; *fimI*; *allD*; *allC*; *allB*; *allR*; *allA*; *allS*; *acrA*; *acrB*; *gtrB*; *nueA*; *msbA*; *sopD2*; *pla*; *hsiB1/vipA*; *hsiC1/vipB*; *mgtC*; *mgtB*; *acrB*; *acrA*; *sopB/sigD*; *pipB*; *ompA*; *sseI/srfH*; *slrP*; *fliS*; *fliG*; *fliI*; *fliM*; *fliN*; *fliP*; *fliQ*; *fliR*; *rcsA*; *tufA*; *sseK1*; *algU*; *sodCI*; *sspH2*; *sspH2*; *tufA*	*acrD*; *comR/ycfQ*; *bhsA/ycfR/comC*; *phoB*; *zinT/yodA*; *kpnO*; *kpnE*; *kpnF*; *kpnO*; *kpnO*; *kmrA*; *dsbB*; *znuA/yebL*; *znuC/yebM*; *znuB/yebI*; *ruvB*; *cutC*; *yfeC*; *rcnR/yohL*; *rcnA/yohM*; *dsbC*; *zntA/yhhO*; *nikR*; *cueP*; *mgtA*; *actP/yjcG*; *arsR*; *arsD*; *arsA*; *arsB*; *arsC*; *silE*; *silS*; *silR*; *silC*; *silF*; *silB*; *silA*; *silP*; *pcoA*; *pcoB*; *pcoC*; *pcoD*; *pcoR*; *pcoS*; *pcoE*; *cutA*; *corD*; *cuiD*; *cutF/nlpE*; *mdtA*; *mdtB*; *mdtC*; *rcnB/yohN*; *zitB/ybgR*; *corC*; *cutE/lnt*; *yqjH*; *zupT/ygiE*; *golS*; *golT*; *oxyRkp*; *fieF/yiip*; *cpxR*; *cpxA*; *dsbA*; *pmrG*; *kpnO*; *cusS*; *cueR/ybbI*; *copA*; *acrD/yffA*; *corA*; *pstS*; *pstC*; *pstA*; *pstB*; *zur/yjbK*; *nfsA*; *kdeA*; *mntH/yfeP*; *acrD/yffA*; *modE*; *modA*; *modB*; *modC*; *kpnO*; *zraP*; *zraR/hydH*; *mntR*; *zntR/yhdM*; *merR*; *merT*; *merP*; *merA*; *merD*; *merE*	*bhpD*; *dcl*	*NoeL*	*AcrIIC1*; *AcrIF7*; *AcrIIA7*

## Data Availability

The whole-genome sequencing data generated in this study are not publicly available due to institutional restrictions but are stored securely by the corresponding author. The data can be obtained from the corresponding author upon reasonable request for research purposes.
